# 

**Published:** 1870-08

**Authors:** 


### INDEX.

**A**

Abbott, Geo., M.D., on Detached Placenta 243
Abscess, Psoas, a case of 106
Abstract of Proceeding of Buffalo Medical Association 18, 48, 88, 218, 297, 378
Action of Urine on the Tissues 59
Address before the Niagara County Medical Society. By A. W. Tyron, M. D. 41
Air in wounds 59
A Lady on Lady Doctors 55
Albany County Medical Society, Annual Meeting. By James S. Bailey, M. D. 131
Albany County Medical Society, proceedings of. Reported by Jas. S. Bailey, M. D. 201, 293, 321, 361, 401
Albany Medical College, complimentary to 73
Albuminuria in Scarlet Fever 234
Alumni University of the Medical Department of the City of New York 319
American Medical Association 278
Amer. Med. Assot'n, meeting of 313
" " " exp. of journey, &c
" " " President of the 434
" " " Proceedings of 380
" " " Recent meeting of 393
" " " Proceedings of, '70 197
Amputation during anæsthesia with Chloral, at the hip joint. By Prof. T. Mack, M. D 250
Anæsthisia, the history of 147
Aneurism, Axillary, by C. C. F. Gay, M.D 102
" of the brachial artery, by C. B. Kibler, M. D 225
Animal Quinine 71
Antiseptic treatment of wounds 32
Application of disinfectants 35
Appointment in St. Peters Hospital, Albany 195
Archives of Ophthalmology and Otology 76
Arrest of Development 158
Arsenic, Soluble Saccharated Oxide of Iron as an antidote to 184

**B**

Babcock, Dr. Heman P., and the Naval Rank 156
Bailey, Jas. S., Report of Annual Meeting of Albany County Medical Society 131
" " " Report of semi-monthly Meetings of Albany County Medical Society 201, 293, 321, 361, 401
" " " on the febrifuge action of cold water used internally 16
Barnes, Dr. Josiah, death of, action of Erie County Medical Association 475
Bell, A. N., M. D., Medical Progress, an oration delivered by 1
Belladonna in the treatment of Typhoid Fever 27
Bellevue Hospital Medical College 192
" " Souvenir of 137
" " Faculty and J. P. White
" " Lectures in 72
Bladder, experiments on the absorbent power of the 68
Blood Stains, detection of 431
Board of Pension Examining Surgeons 317
Bone, hydatids in 63
Books & Pamphlets Received, 40, 80, 120, 160, 199, 240, 280, 320, 360, 400, 440, 480
Bradnack, F., reports of clinical remarks by Prof. Thos. F. Rochester, M. D., 124 167, 227, 300
Breasts, a woman with four, 147
Bromide of Potassium 139
Bromide of Potassium, correcting influences of, on Opium 346
Bronchitis, asthmatic, Chloral in 270
Buffalo, as a field for physicians 235
" Dispensaries 73
Buffalo Medical and Surgical Journal 469
Bloodvessels, minute, report and photomicroscopic illustrations 152

**C**

Carbolic Acid in the treatment of Uterine Catarrh by internal application 31
" Poisonous effects of 57
" Preparations 145
Carbonic Acid, physiological action 180
Chloral, the poisonous dose of 30
" Strichnia as an antidote to 30
" Amputation during Anæsthesia with 88
" influence in parturition 140
" death from, by Wm. Holbrook, M. D 257
" in asthmatic bronchitis 270
Chloroform, by C. A. Lee, M. D 179
" danger from inhalation of 268
" and morphia, combined action of 108
Clinical Remarks by Prof. J. F. Miner, M. D., on surgical cases occurring in the Buffalo Hospital of the Sisters of Charity, reported by W. W. Miner, A. B., on Pterygium 104
" Granular lids 104
" Amputation of foot 105
" Injury of the eye 105
" Hip-joint disease 127
" Resection of bone after amputation 130

ClinicalAmputation of the knee joint .... 172
" Encephaloid cancer .......... 174
" Bubo ....................... 174
" Enucleation of the eye-ball.. 175
" Hydrocele ................. 229
" Transplantation of skin, 230, 328
" Prolapsus ani ............... 258
" Club Foot .................. 260
" Exostosis of right innominatum ........................ 329
Clinical Remarks by Prof. Thos. F. Rochester, M. D.. in Hospital Sisters Charity, reported by F. Bradnack. On Diptheria.... 124
" Lead Colic ................... 125
" Intermittent Fever ........... 167
" Pleurisy ..................... 170
" Œdema Glottitis .............. 227
" Pneumonia .................... 255
" Pulmonary Tuberculosis ....... 300
Collegiate Honors ...................... 25
Commencement Exercises at the Buffalo Medical College, names of graduates, &c 275
Connection between inflammatory connection of the uterus and its displacement ................................ 271
Constipation, chronic, therapeutics of .. 68
Coroners Inquests and John Hauenstein, 301, 309
Crothers, T. D., M. D., on Therapeutic power of oxygen gas ................ 163
Croup, local treatment of .............. 107
Cundurango, a new cure for cancer ...... 394

**D**

Death of Prof. E. H. Elliott ............ 278
" Dr. Gorham F. Pratt, resolutions of Erie Co. Medical Society ....... 353
" Barent P. Staats, M. D., of Albany ............................ 474
" Dr. Barnes, action of Erie County Medical Society .............. 475
" Dr. T. T. Lockwood, resolutions on the ......................... 477
Dematophytæ, notes on the .............. 65
Development, arrest of ................ 158
Diagnosis, rectal, in women ............. 61
Diarrhea, chronic, compound solution of Iodine in, by E. L. Shurley, M. D. ..... 302
Disease, local causes of ................. 71
" Special, from mental strain ...... 67
" Stimulants in the treatment of .. 115
Dropsy, ovarian, hypodermic syringe in diagnosing ........................ 111
Drug Clerk Law ........................ 399

**E**

Early symptoms and treatment of Pott's disease of the spine ............... 112
Earth closet system ..................... 398
Eastmam, Prof. H. N., M. D., on quinine in Croup ........................... 207
Efficient and simple substitute for the stomach pump ........................ 59
Elephantiasis Arabum, a case of, by H.D. Ingraham, M. D. ................... 225
Elliott, Prof. E. H., death of ........... 278
Erie County Medical Society, semi-annual meeting of ......................... 435
Errata ................................ 199
Errors in Dr. Gay's Hospital Notes on Hydrocele .......................... 476
Ethical and Scientific Unity in the Medical Profession. An address before the graduates of the medical department of tne University of Buffalo, of the class of 1871. By Prof. E. M. Moore, M. D.. 281
Eustachian tube, chronic spasm of the muscles of the ......................... 272
Exchanges, list of ...................... 199
Extension is hip-disease ................ 139

**F**

Febrifuge action of cold water used internally, by Jas. S. Bailey, M. D. ...... 16
Femur, dislocation and fracture of head of. By L. A. Harcourt, M. D. ......... 241
Fever Germs in milk ................... 142
Flint, Austin, M. D., extract from a paper upon the pathological relations of the gastric and intestinal tubes ........ 304
Fracture of the 7th cervical vertebra. By J. W. Grosvenor, M. D. ................ 415

**G**

Gastric and intestinal tubes, extract from a paper upon the pathological relations of the. By Austin Flint, M. D. ....... 304
Gay, C. C. F., M. D., on laceration of the perinium ............................ 52
" Axillery aneurism ............ 102
" On retention of urine caused by traumatic stricture ......... 176
" Varicose veins ............... 178
" Amputation of the thigh ...... 177
" Removal of mammary gland for schirrhus disease ........... 252
" Operation for radical cure of hernia ..................... 253
" Necrosis ..................... 253
" Operation and radical cure of hydrocele ................. 412
Gibbs, O. C., M. D., on speedy and spontaneous recovery from rupture of rectum and bladder ..................... 161
Grosvenor, J. W., M. D., a case of fracture of the 7th cervical vertebra ...... 412

**H**

Hæmorrhage, anæmia, and emphysema in the lungs, by injuries to the base of the brain ................................ 427
Hamburg canal as an odorant and a beverage ................................ 352
Harcourt, L. A., M. D., on obstetrical practice and mal-peactice .... 165
" A case of dislocation and fracture of the head of the femur 241
Harrington, D. W. Report of surgical cases treated in the Buffalo General Hospital during service term of J. F. Miner, M. D ................................ 233
Hauenstein, John, M. D., on coroners inquests ............................ 301
" Verdict of coroners inquests ... 333
Hip disease, extension in .............. 131
History of anæsthesia .................. 147
Holbrook, Wm., M. D., a case of death from chloral .......................... 257
Howard University, first commencement of the ............................... 279
Hydatids in bone ....................... 63

**I**

Influence of non-specific eminations on the public health .................... 340
Ingraham, H. D., M. D., on a case of elephantiasis arabum .................. 225
Is it right to vaccinate or re-vaccinate pregnant women? .................. 431
Items ................................. 432
" Personal ........................ 195
" Selections and remarks. By W. W. Miner, A. B. ................ 74, 117, 193

**J**

Jewett, Harvey, M. D., on treatment of Puerperal Eclampsia .................. 441
Joints, clinical remarks on wounds into .. 267

L

Lady doctors, a lady on 55
Lectures in the Bellevue Hospital Medical College 272
" introductory, in the Buffalo Medical College 120
Lee, C. A., M. D., on chloroform 179
Life, nature of 437
List of Exchanges 200
Literary exchanges, notices of 196
Liver the seat of formation of urea 266
Local causes of disease 71
Lockwood, Dr. T. T., death of, action of Erie County Medical Society 477
Long Island Medical College Hospital, a card from 158
Lymphatic system of the human body, by Z, C. McElroy, M. D 449

M

Mack, Prof. T., on amputation at the hip joint 250
Massachusetts Medical Society 151
McElroy, Z. C., M. D., report of committee on new remedies 81
" On the lymphatic system of the human body 449
Medical Journal, a new 76
" " Association, proceedings of the 389
" Prescriptions 141
" Progress, an oration delivered by A. N. Bell, M. D 1
" Society of the District of Columbia 237
" Society of Monroe County, annual meeting of 456
" Society of N. Y, State, meeting of 238
" Society of N. Y. State, sixty-fifth meeting of the 272
" Society of N. Y. State, transactions of the 319
Medicines, medical men and quacks, newspaper notices of 433
Mental strain, special diseases from 67
Michigan University, appointment in, and admission of women 479
Miner, Prof. J. F., M. D., notes on interesting obstetrical cases 121
" On clinical remarks of surgical cases occurring in the Buffalo Hospital of Sisters of Charity, reported by W. W. Miner, A. B., 104, 105, 127, 130, 172, 174, 175, 229, 230, 328, 258, 260, 329
Miner. W. W., reports of clinical remarks of surgical cases occurring in the Buffalo Hospital of Sisters Charity, by Prof. J. F. Miner, M. D., 104, 127, 172, 229, 258
Milk, fever germs in 142
Moore, Prof. E. M., M. D., Ethical and Scientific Unity in the Medical Profession. An address before the graduates of the Medical department of the University of Buffalo, of class of 1871 281
Morphia and chloroform, combined action of 108

N

Nature of life 437
Naval Rank 394
" " and Dr. H. P. Babcock 156
" Staff Question 263
Nervous system, syphilis of the 183
Nitrous oxide gas in surgery 58

O

Obstetrical cases, notes on by J. F. Miner, M. D., 121
Obstetrical practice and mal-practice, by L. A. Harcourt, M. D. 165
Œdema, production of 181
Open air treatment of phthisis 63
Ophthalmology and Otology, archives of 76
Opium, corrective influence of bromide of potassium on 346
Ovarian dropsy, hypodermic syringe in diagnosing 111
Ovariotomy, attempted 270
Oxide, soluble saccharated, of iron as an antidote to arsenic 184
Oxygen gas, synopsis of a paper on the therapeutic power of, by T. D. Crothers, M. D., 163

P

Parturition, influence of chloral in 140
Pathology and treatment of scarlet fever 106
" of tuberculosis, remarks on the 186
Pension bureau and Dr. Spooner 435
" Examining Surgeons, board of 317
" " " report of special committee on the claims of irregular practitioners for places as 335
Perineum, laceration of the, by C. C. F. Gay, M. D. 52
Phthisis, Open air treatment of 63
Physicians, Buffalo as a field for 235
Physiological action of carbolic acid 180
Photo-microscopic illustrations and report of the histology of minute blood-vessels 152
Placenta, detached, by Geo. Abbott, M.D. 243
" Praevia 569
Poisonous effects of carbolic acid 57
Pool of Siloam 395
Potts' disease of the spine, early symptoms and treatment of 112
Pratt, Dr Gorham F, death of, action of Erie County Medical association 353
Pregnant women, is it right to vaccinate and re-vaccinate 431
Prescriptions, medical 146
" percentage on 238
Prolapsus Uteri, treatment of 309
Propylamin in rheumatism
Puerperal Eclampsia, by Harvey Jewett, M. D., 441
" Fever, cases of 109

Q

Quackery, transcendental 150
Quacks, medical men and medicines, newspaper notices of 433
Quinine 422
" Animal 71
" in croup, by Prof. H. N. Eastman, M. D. 207

R

Rectal Diagnosis in Women 60
Rectum and Bladder, speedy and spontaneous recovery from rupture of, by O. C. Gibbs, M.D. 161
Red Blood Corpuscles, structure of 421
Relapsing Fever 143
Remedies, report of committee on new, by Z. C. McElroy, M. D 81
Rheumatism, propylamin in 417
REVIEWS.—Report of the Metropolitan Board of Health 35
Facts and remarks concerning idiocy, by Edward Seguin, M. D. 37

History of nine cases of Ovariotomy, by Prof. H Gaillard Thomas, M. D. 37
Specialism in its relations to Practical Medicine, by G.S. Hubbard, M.D 38
Medico-Legal Study of the case of Daniel McFarland 38
Report of the Committee on the relations of alcohol to medicine By John Bell, M. D., Chairman 39
Medical opinion of Charles A. Lee Case of Carlton Yates 40
DaCosta's Medical Diagnosis 77
Medicine and Morals. By J. A. Mowris, M. D 77
The Preventive Obstacle, by L. F E. Bergeret, M. D. 79
Life at Home, by Wm. Aikman, D. D. 79
On the Sedative Action of Calomel, by F. D. Lente, M. D 79
Treatment, Cleft Palate, by W. R. Whitehead, M. D. 79
Case of the People against E B. Fero, by C. H. Porter, M D 80
Pathology of Bright's Disease, by W. B. Lewis, M. D. 80
Physiological action of Acidum Phosphoricum Dilutum, by J. B. Andrews, M. D. 80
Three Cases, Imperforate Anus, by J. H Pooley, M. D. 80
Tanner's Practice of Medicine 119
Byford's Theory and Practice of Obstet rics 119
Basham on Renal Disease 119
Physician's Visiting list for 1871 130
Physical Exploration of the Rectum, by Wm. Bodenhamer, A. M., M. D. 159
Transactions of the America Medical Association 197
Hand-Book of Medical Microscopy, by Joseph Richardson, M. D. 198
Bumstead on Venereal Diseases 239
Heath's Practical Anatomy. Edited by W. W. Keen, M. D. 239
Galvano-Therapeutics, by Wm. B. Neftel, M. D. 239
Satan in Society 279
Bound volumes of the Gynæcological Society of Boston 319
Physics and Physiology of Spiritualism, by Wm. A. Hammond, M. D. 320
Diseases of the Spine and Nerves, by C B. Ratcliffe, London 354
A Treatise of Physiology andHygiene by J. C. Hutchinson, M. D, 354
Health Officer's Annual Report of the city of Rochester, by B. L. Hovey, M. D. 355
Malpractice Suit of Walsh vs Sayre 356
Medical and Surgical Memoirs, by Joseph Jones, M. D 357
Treatment of Croup, by F Barker, M. D. 358
Guide to Examination of Urine, by J. W. Legg, M D 358
Circular Nos. 3 and 4, War Department Surgeon General's Office 358
Report of the board of health, Chicago 359
Retention of Urine dependant on Stricture of the Urethra, by A. Stein, M. D 359
Photographic Review of Medicine and Surgery 359
Memoirs U. S. Sanitary Commission 395
Causation and Treatment of Reflex Insanity in Women, by H. R. Storer, M. D 396
Freedmen Trial, by David Dimon 397
Report of Michigan Asylum for Insane 397
Anæsthetics, by Edward R Squibb 397
Management of Obstetrical Forceps, by C. C. P. Clark, M. D. 397
Report N. Y. State Lunatic Asylum for Insane Criminals 398
Valedictory Address, by Prof. M. Gunn, M. D, Chicago 398
Chemistry, General, Medical and Pharmaceuticia, by J. Attfield, M. D, F. C. S. 437
Wasting Diseases of children, by Eustace Smith, M. D 438
Change of Life, by E John Tilt, M.D. 538
Insanity and its taeatment, by G. Fielding Blandford, M. D. 439
Medical Section of the work of N.P. Dou beveyer, by Dr. Gans 439
Annual Report of Commissioner of Quar antine 439
Dactylitis Syphilitica, with observations on Syphilitic Lesion of the joints, by R. W. Taylor, M D 439
Uterine Catarrh frequently the cause of Sterility, by H. E. Gantillon, M.D 440
The Study of Dermatology, by Louis A. Duhring, M. D 440
The eye in health and disease, by B. Joy Jeffries, A. M., M. D 478
Physiological effect on muscular exercise, by Austin Flint, Jr., M. D 479
Hæmatoma Auris, by E. R. Hun, M D 440
Rightmire, Geo., M. D, on a case of inverted uterus 330
Rochester, Thos. F., Clinical remarks on cases occurring in Buffalo Hospital of the Sisters of Charity, reported by F. Bradnack, 124, 125, 167, 170, 227, 255, 300

### S

Scarlet Fever, albuminuria in 234
" " pathology and treatment of 106
Shurley, E. L., M. D., Compound solution of Iodine in chronic diarrhea 302
Souvenir of Bellevue Hospital Medical College to Prof. J. P. White, M. D. 237
Spasm, chronic, of the muscles of the Eustachian tube 272
Spooner, Dr., and the Pension Bureau 435
Staats, Barent P., death of 474
Stimulants in the treatment of disease 115
St Peter's Hospital Albany, appointment in the 195
Stomach Pump, simple and efficient substitute for the 59
Structure of the red blood corpuscles 421
Strychnia as an antidote to chloral 30
Subcutaneous division of the thigh bone 70
Suits for mal-practice 359
Surgical cases treated at the Buffalo General Hospital, by C. C. F. Gay, M.D, 176, 252
Surgical cases treated at the Buffalo General Hospital during service term of Dr. J. F. Miner, reported by D. W. Harrington, acting house physician 233
Surgery, American, in Paris 352
" Nitrous oxide gas in 58
Simpson, Sir Ja's Y, posthumous works of 359
Syphilis of the nervous system 183

### T

Tendon, divided, re-production and reunion of 65
Tetanus treated by woorali, calaber bean and chloral hydrate 267
Therapeutics of chronic constipation 68
Thigh bone, subcutaneous division of the 70

Thoracentesis ........................... 111
Tonsils, enlarged, treatment of ........... 431
Tyron, A. W., M. D , address before the Niagara County Medical Society ....... 41
Typhoid Fever, belladonna in the treatment of ..................... 27
" " treatment of, by cold water baths ..................... 349

### U

Ulcer, simple, of the stomach, by Prof. H. Ziemssen, translated by Geo. Niemeier, M. D .... .....................331, 369
United States Sanitary Commission, memoirs of ............................. 395
University of Wooster .................. 359
Urea, the liver the seat of the formation of ................................ 266
Uterine Catarrh, treatment of, by the application of carbolic acid ............ 31
Uterus, and its displacements, connection between inflammatory connections of the ..................................... 271
Uterus, inverted, a case of, by Geo. Rightmire, M. D .......................... 330

### V

Verdict of coroner's inquests, by John Hauenstein, M. D ...................... 333

### W

Water Baths, cold, in the treatment of Typhoid Fever ......................... 349
Will snake poison kill a snake? ........... 430
White, Prof. J. P., souvenir of Bellevue Hospital Medical College to ..... 237
" and Faculty of Bellevue Hospital Medical College ...............
Woman, a, with four breasts .............. 147
Wounds, antiseptic treatment of ......... 32
" Air in ........................... 59
" into joints, clinical remarks on. 267

### Y

Yellow Fever ............................ 399

### Z

Ziemssen, Prof. H.. on the treatment of simple ulcer of the stomach, translated by Geo. Niemeier, M. D., ............331, 369

*Gynaecological Society of Boston*:

We have received the By-laws, Constitution List of Officers. etc., etc., of this society. It is plain to see, by its monthly journal, its valuable contributions and reports, and by its general activity and prosperity that this organization is, and is to become a great power, situated at the "hub," which will make its influence felt for progress and reform.

---

### Books and Pamphlets Received.

Dr. Costa's Medical Diagnosis.—Third edition.

Archives of Ophthalmology and Otology.—Vol. 1, No. 2.

Theory and practice of Obstetrics. By William H. Byford, M. D.

The Physical Exploration of the Rectum. By William Bodenhamer, M. D.

Life at Home. By William Aikman, D. D.

Basham, on Rectal Diseases.

Report to the Faculty of the Medical Department of the University of Louisiana, in regard to the Convention of Medical Teachers, lately held in Washington City.

Saint Louis College of Physicians and Surgeons.

Health Officer's Annual Report of the City of Rochester, for the year ending March 31, 1870. By Bleeker L. Hovey.

Harvard University.—Eighty-seventh Medical course.

Purulent Otitis Media, caused by the Nasal Douche, and accompanied by Double Hearing. By H. Knapp.

University of Michigan.

Hæmatoma Auris. By E. R. Hun, M. D.

The Origin of Diabetes, with some new experiments regarding the Glycogenic Function of the Liver. By W. T. Lusk, M. D.

